# Effect of a Behavioral Therapy-Based Virtual Reality Application on Quality of Life in Chronic Low Back Pain

**DOI:** 10.1097/AJP.0000000000001110

**Published:** 2023-04-01

**Authors:** Tjitske D. Groenveld, Merlijn L.M. Smits, Jesper Knoop, Jan Willem Kallewaard, J. Bart Staal, Marjan de Vries, Harry van Goor

**Affiliations:** * Radboud University Medical Center, Department of Surgery, Radboud Institute for Health Sciences; ‡ Hogeschool Arnhem en Nijmegen University of Applied Science, Research Group Musculoskeletal Rehabilitation, Nijmegen; † PBLQ, The Hague; § Rijnstate Hospital, Department of Anesthesiology, Arnhem, The Netherlands

**Keywords:** virtual reality, chronic low back pain, behavioral therapy, biopsychosocial treatment, health-related quality of life

## Abstract

Low back pain is the leading cause of years lived with disability with a large impact on quality of life and resistance to a broad array of current treatments. This study aimed to investigate the effect of a novel self-administered behavioral therapy-based virtual reality (VR) application on the quality of life of patients with nonspecific chronic low back pain (CLBP).

**Methods:**

A pilot randomized controlled trial was conducted in adults with nonspecific CLBP with moderate to severe pain, waiting for treatment in a teaching hospital-based pain clinic. The intervention group used a self-administered behavioral therapy-based VR application for at least 10 minutes daily for 4 weeks. The control group received standard care. The primary outcome was quality of life at 4 weeks measured by the short form-12 physical and mental scores. Secondary outcomes were daily worst and least pain, pain coping strategies, activities of daily living, positive health, anxiety, and depression. Discontinuation of therapy and adverse events were analyzed as well.

**Results:**

Forty-one patients were included. One patient withdrew due to personal reasons. No significant treatment effect was found for the short form-12 physical score (mean difference: 2.6 points; 95% CI: −5.60 to 0.48) and mental score (−1.75; −6.04 to 2.53) at 4 weeks. There was a significant treatment effect for daily “worst pain score” (*F* [1, 91.425] = 33.3, *P* < 0.001) and “least pain score” (*F* [1, 30.069] = 11.5, *P* = 0.002). Three patients reported mild and temporary dizziness.

**Discussion:**

Four weeks of self-administered VR for CLBP does not improve quality of life, however, it may positively affect daily pain experience.

## INTRODUCTION

Low back pain is the leading cause of years lived with disability, accounting for over 10% of total years lived with disability.^1^ Up to half of patients with low back pain develop chronic pain (ie pain, which lasts for ≥3 mo).^2^ In Europe, as many as 1 in 5 people report chronic low back pain (CLBP).^3^ CLBP has a large negative impact on quality of life in terms of physical and mental health.^4^

Treatment for CLBP consists mainly of physiotherapy and analgesics, in particular nonsteroidal anti-inflammatory drugs (NSAIDs) and opioids.^4^,^5^ Results of this combined treatment are disappointing with a pain improvement of 15 points (scale: 0 to 100) and an improvement of functional limitations of 7 points (0 to 100) in the majority of patients.^5^,^6^ Moreover, long-term use of NSAIDs and opioids is associated with serious side effects, for example, gastrointestinal bleeding and arterial thrombosis and addiction, and mental dysfunction.^7^,^8^ Low success of this physically oriented treatment can be understood when considering the current knowledge of the role of central sensitization in chronic pain disorders, which is observed as an amplification of neural signaling within the central nervous system that elicits pain hypersensitivity.^9^ Together with the knowledge about psychosocial risk factors for chronic pain, such as pain catastrophizing, passive coping strategies, and fear-avoidance beliefs, this has led towards programs for a more holistic CLBP treatment and multidisciplinary biopsychosocial rehabilitation.^10^–^13^ Besides physical exercises and analgesics, pain education and self-management are core elements of these programs, that enable patients to understand their condition and to improve their pain beliefs and coping strategies. Multiple other nonpharmacological interventions are being combined in the biopsychosocial approach of CLBP, including cognitive-behavioral therapy (CBT), mindfulness-based interventions, and to a lesser extent hypnotherapy and eye movement, desensitization, and reprocessing therapy.^11^,^14^ Although there is an international consensus about this multidisciplinary biopsychosocial approach, there seems to be a “know-do” gap, with therapists and physicians struggling to apply the psychosocial components of CLBP treatment.^12^ Possible explanations are the complex organization of multidisciplinary treatment, shortage of personnel, and lack of commitment by patients. Another problem is the generally low adherence to prescribed treatments in patients with CLBP, with adherence rates varying from 50% to 70%.^15^ Barriers mentioned by patients are, for example, lack of motivation, burden of exercising, and negative attitudes towards chronic pain.^16^

The use of a behavioral therapy-based virtual reality (VR) intervention might provide therapists and physicians with the means to seamlessly incorporate the psychosocial aspects of pain into the treatment. VR consists of a head-mounted device immersing the user in a 3-dimensional virtual world, which can be used to distract patients from experiencing pain and anxiety and to treat psychological disorders.^17^,^18^ Moreover, VR can be applied by patients at home, thereby facilitating treatment adherence. In contrast to analgesics, VR has only a few and usually mild side effects, such as dizziness and nausea.^19^ A recent systematic review showed the effectiveness of VR on chronic pain in general, but especially for patients reporting an a numeric rating scale pain score of 4 and higher.^20^ In a randomized controlled trial, Garcia et al^21^ showed a significant reduction of average pain intensity and pain-related interference with activity, mood, and stress, treating patients with CLBP at home with an 8-week VR pain program, containing modules for pain education, distraction, and mindfulness. It is argued that the function and quality of life of patients with chronic pain should be prioritized as an outcome measure of VR in CLBP over pain relief.^22^ The current study focuses on the effect of a novel VR application on the health-related quality of life of patients with nonspecific CLBP. This VR application combines patient education that explains the maladaptive changes in the central nervous system with elements of several evidence-based cognitive-behavioral pain therapies into one integrated application.^11^,^14^,^23^

## METHODS

### Design

This was a pilot open-label randomized controlled trial conducted during the coronavirus disease (COVID) pandemic between January 2020 and January 2021 at Rijnstate Hospital, Arnhem, the Netherlands. The results of the qualitative part of this study have been reported earlier.^24^ Ethical approval was obtained by the Research Ethics Committee of the Radboud University Medical Centre and Rijnstate Hospital in Nijmegen and Arnhem, the Netherlands (CMO Arnhem-Nijmegen, NL70042.091.19). This trial was conducted according to the principles of the Helsinki Declaration and in accordance with Dutch guidelines, regulations, and Acts (Medical Research involving Human Subjects Act, WMO). The trial was registered at ClinicalTrials.gov (NCT04042090). The trial is reported after the CONSORT statement for randomized trials.

### Participants

The study population comprised patients of 18 years and older with nonspecific CLBP reporting an average pain score of 4 and higher on an 11-point Likert scale in the week preceding enrollment. Chronic pain was defined as pain lasting for at least 3 months. Other inclusion criteria were: (1) the patient has an estimated waiting period of at least 6 weeks on the day of recruitment on the waiting list; (2) the patient is not yet receiving treatment, apart from analgesics or physiotherapy; (3) the patient did not receive any invasive treatment for his chronic nonspecific low-back pain in the last year; (4) patients read and understand the Dutch language. Exclusion criteria were: (1) the patient has radicular pain that is worse than the CLBP; (2) inclusion in another trial to evaluate new ways of treating pain; (3) the presence of severe anxiety or depression; (4) not being able to handle VR due to delirium, dementia, epilepsy, severe hearing/visual impairment, skin of the head or face not intact, and high risk of Meticillin-resistant Staphylococcus aureus. Patients were recruited from a waiting list for advanced pain treatment in a pain clinic of a large teaching hospital. No sample size calculation was performed for this pilot study, but we considered 20 in each group to be sufficient for our primary study aims to investigate the preliminary effectiveness of VR on health-related quality of life in this population.

### Intervention

A novel application of VR for chronic pain management (Reducept) was used with an Oculus Go (Facebook Technologies, LLC) head-mounted display. This therapeutic application, still investigational, was designed in cocreation with patients, psychologists, educationalists, and software developers. It is based on the biopsychosocial model of pain, which describes the pain as the result of a combination of physical, psychological, and social factors.^25^ The application is designed as a game, in which a patient “travels” through the nervous system sitting in a pod. During the journey, a calm voice explains the mechanisms of pain, which are made visual in the virtual environment. Along the way, patients are offered 5 different games, each containing exercises based on different psychological treatment principles (ie, acceptance and commitment therapy, mindfulness, hypnotherapy, eye movement, and desensitization and reprocessing) incorporating an education element, explaining the maladaptive changes in the central nervous system, in every game (Appendix A, Supplemental Digital Content 1, http://links.lww.com/CJP/A951). Each game takes about 10 minutes per session, and the different behavioral therapy principles are alternated over the days. The application is designed for patients to use independently at their homes. The VR group used VR while on the waiting list for a follow-up visit to discuss advanced pain treatment. The first VR session was performed under research staff supervision at the patient’s home. Patients were instructed to use the VR intervention for at least 10 minutes daily for 4 weeks with the restriction of 30 minutes per session and 3 sessions per day. The control group was also on the waiting list for a follow-up visit to discuss advanced pain treatment, but received no additional treatment or VR intervention.

### Study Procedures

The treating physician (J.W.K.; anaesthesiologist and pain physician) identified eligible patients, provided verbal and written information, and subsequently obtained written informed consent. Patients were randomly assigned (1:1) to the VR or control group using computer-generated block randomization. Randomization was performed by the research staff, the treating physician was blinded for allocation at the moment of inclusion.

All patients were approached by the research staff and asked to fill out questionnaires at baseline, at the end of treatment at 4 weeks, and at 4 months follow-up. Furthermore, patient characteristics were obtained at baseline for analysis of associations with VR effectiveness. Patients were asked to keep a diary of daily pain scores and analgesic use during the intervention period. On days 7 and 14, telephone interviews were carried out to monitor adherence, adverse events, and technical problems. The research staff was around the clock available for questions by telephone.

### Outcome Measures

Outcome measures were chosen according to the core outcome measures for chronic pain as recommended in the IMMPACT guidelines.^26^

#### Primary Outcome

The primary outcome was quality of life measured by the short form-12 (SF-12) at 4 weeks.^27^ The SF-12 is validated in patients with CLBP and includes a physical and mental domain, for which a norm-based score is calculated between 0 and 100 points using the method described by Ware.^28^

#### Secondary Outcomes

Secondary outcomes were SF-12 at 4 months, daily pain scores and analgesics use, and the questionnaires at 4 weeks and 4 months as described. Another outcome was feasibility in terms of acceptability, usability, and tolerability.^29^

Pain scores comprised pain scores (0 to 100 mm) on “worst pain experienced today” and “least pain experienced today” (0 = no pain, 10 = extreme pain), an 11-point visual analog scale (VAS) measuring the percentage of time having experienced severe pain (0% = none of the time, 100% = all the time), and an 11-point Likert scale measuring, to which extent pain has prevented the patient from moving in bed, taking a deep breath or coughing, and sleeping (0 = no influence, 10 = completely prevented).^26^ Further, patients registered which analgesics they used daily. The number of patients taking analgesics at least once per study week was counted per category (ie, paracetamol, NSAIDs, or opioids). Opioids comprise weak opioids, including tramadol and codeine, and strong opioids, including (derivatives of) morphine, fentanyl, and oxycodone.

The Pain Catastrophizing Scale and the pain coping and cognition list were used to evaluate pain catastrophizing and pain coping strategies.^30^,^31^ The pain interference score, derived from the brief pain inventory, was used to measure the influence of pain on activities of daily living.^32^ The Oswestry Low Back Pain Disability Index was used to measure the degree of functional disability because of low back pain.^33^ The independence of patients regarding activities of daily living was measured using the Nottingham Extended Activities of Daily Living questionnaire (NEADL).^34^ The Positive Health Questionnaire was used to measure the patient’s feelings about their overall health and about the dimensions of “bodily functions”, “mental well-being”, “meaningfulness”, “quality of life”, “participation”, and “daily functioning.”^35^ The hospital anxiety and depression scale (HADS) was used as a global measure of psychological distress.^36^

#### Feasibility

Discontinuation of VR was registered as an acceptability outcome, together with reasons for withdrawal. Adherence was monitored through weekly telephone interviews. Adverse events were registered as a tolerability outcome through open-ended questions during the weekly telephone calls.

### Statistical Analyses

All analyses were done using IBM SPSS Statistics 25. Outcomes were analyzed following the intention-to-treat principle. Descriptive statistics were used for presenting baseline data, adverse events, and feasibility outcomes.

For primary efficacy analyses, linear mixed models were used to analyze the effectiveness of the VR intervention at 4 weeks, with “treatment,” “time,” and “treatment time” as fixed effects. A within-person correlation was accounted for by using a repeated measure design. Baseline values were incorporated as a covariate. Possible other covariates were added on a rational basis and only retained when having a significant effect on the model. The best-fitting covariance structure was determined using likelihood ratio tests. Maximum likelihood estimation was used to be able to perform these likelihood ratio tests. For the final model, the restricted maximum likelihood was used, since this is recommended for smaller sample sizes. Residuals were checked for normality. An alpha of 0.05 was considered significant.

All time points (postintervention at 4 wk and follow-up at 4 mo) were included in a linear mixed models analysis with repeated measure design. If a significant difference was detected, a pairwise comparison per time point was performed. Pain scores were analyzed using linear mixed models following the same methods. Other secondary outcomes were analyzed using descriptive statistics. When possible differences were observed, delta scores between baseline and at 4 weeks were assessed using *t* test or Mann-Whitney test depending on the distribution. To explore the effect of VR on the elderly compared with younger participants, the correlation between age and delta scores of SF-12 physical and mental were assessed.

## RESULTS

**FIGURE 1:**
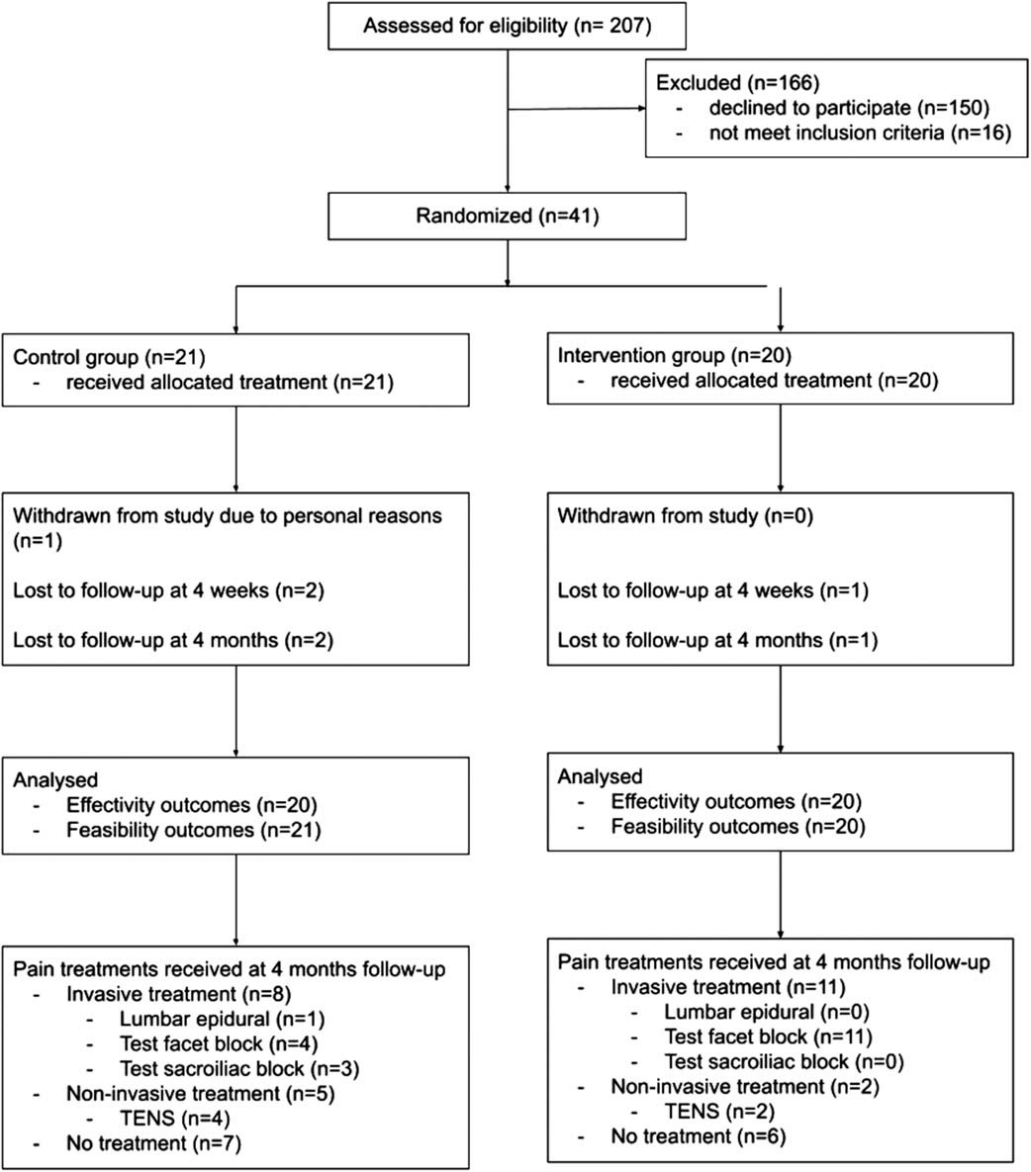
Study flowchart. TENS indicates transcutaneous electrical nerve stimulation.

A total of 207 patients were assessed for eligibility and 41 patients were included between January 2020 and January 2021 (Fig. 1). Patients not meeting inclusion criteria (n = 16) had leg pain rather than low back pain. One patient withdrew from the control group because of personal reasons. In total, 6 patients did not complete the questionnaires and were considered lost to follow-up. The mean age (SD) was 51.5 (11.9) years; 83% were females. Baseline characteristics were comparable between groups (Table 1).

**TABLE 1:** Patient Characteristics

	Intervention (n = 20); n (%)	Control (n = 20); n (%)
Age (y)		
Mean (SD)	51 (2.9)	52 (2.5)
Range	27-71	30-73
Sex (F)	17 (85)	16 (80)
Pain duration (y)		
Median (Q1; Q3)	5 (2; 14)	4 (1; 11)
Range	0.2-30	0.5-30
ISCED		
0-1—primary	1 (5)	1 (5)
2—lower secondary	3 (15)	5 (25)
3-4—upper secondary	9 (45)	7 (35)
5-7—third level	5 (25)	7 (35)
Missing	2 (10)	0
Work status		
>30 h per week	6 (30)	5 (25)
<30 h per week	2 (10)	4 (20)
Retired	1 (5)	2 (10)
Sickness benefit	2 (10)	2 (10)
Incapacitated for work	3 (15)	2 (10)
Other	4 (20)	5 (25)
Missing	2 (10)	0
Daily use ≥3 digital devices	16 (80)	15 (75)
Experience VR	3 (15)	5 (25)

ISCED 0-1: early childhood to primary education.

ISCED 2: lower secondary.

ISCED 3-4: upper secondary to postsecondary nontertiary education.

ISCED 5-7: short-cycle tertiary education, bachelor's, master's, or equivalent level.

ISCED indicates International Standard Classification of Education; VR, virtual reality.

In the VR group, 1 patient received pulsed radiofrequency stimulation and 2 patients received a transcutaneous electrical nerve stimulator within the 4-week intervention period. No patients in the control group received advanced pain treatment during the intervention period. Eleven patients in the VR group and 8 patients in the control group received advanced pain treatment between the end of the VR intervention at 4 weeks and 4 months follow-up (Fig. 1).

### Primary Outcome

**FIGURE 2:**
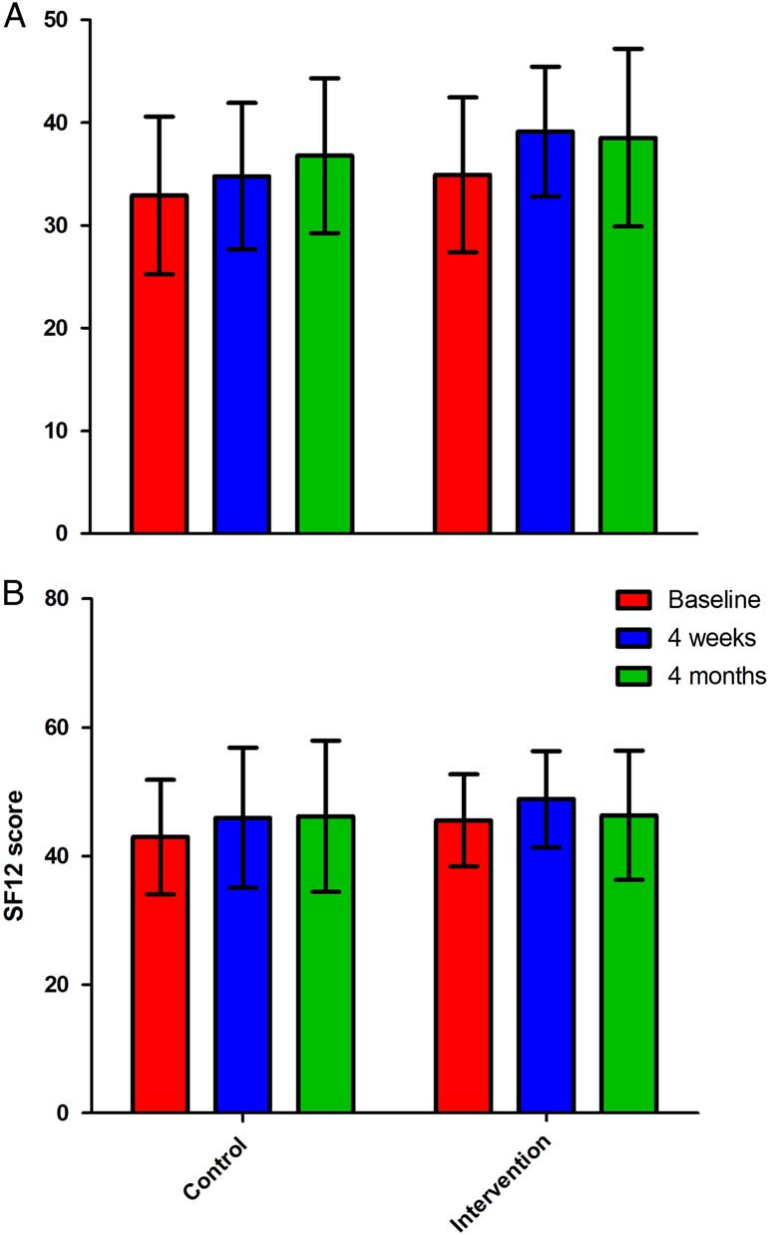
SF-12 physical (A) and mental (B) scores, mean and SD. SF-12 indicates short form-12.

Physical and mental scores of the SF-12 are shown in Figure 2. Estimated marginal means for SF-12 physical and mental are reported in Table 2. SF-12 scores at 4 weeks did not significantly differ between the groups for the physical subscale (mean difference (95% CI): −2.56 (−5.60 to 0.48); *P* = 0.96) or the mental subscale (−1.75 (−6.04 to 2.53); *P* = 0.41).

**TABLE 2:** EMMs (95% CIs) of SF-12 Physical and SF-12 Mental at 4 Weeks and 4 Months

	EMM at 4 wk		EMM at 4 mo	
	VR	Control	VR	Control
SF-12 physical	38.2 (36.1-40.3)	35.6 (33.4-37.8)	37.6 (34.6-40.5)	38.4 (35.1-41.6)
SF-12 mental	48.4 (45.4-51.3)	46.6 (43.5-49.7)	46.4 (43.0-49.9)	46.0 (42.2-49.8)

EMM indicates estimated marginal mean; SF-12, short form-12; VR, virtual reality.

No significant main treatment effect was found between groups over time for the SF-12 physical subscale (*F* (1, 22.943) = 0.380, *P* = 0.54) and mental subscale (*F* (1, 27.995) = 0.347, *P* = 0.56). No significant treatment-time interaction was found for the SF-12 physical subscale (*F* (1, 26.064) = 2.158, *P* = 0.15) and the mental subscale (*F* (1, 30.205) = 0.227, *P* = 0.64). No covariates, including baseline pain score, HADS, opioid use, and treatment during the intervention period, had a significant effect on the models for the physical and mental subscales and, therefore, none were retained in the models.

### Secondary Outcomes

**FIGURE 3:**
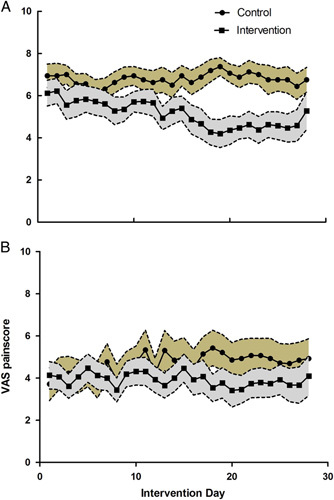
Mean and standard error of the mean of daily worst VAS pain score (A) and daily least VAS pain score (B) during the intervention period. In the intervention group, data were missing for 1 patient from day 8, for 1 patient from day 12, for 1 patient from day 13, for 2 patients from day 25, for 1 patient from day 26, and for 1 patient from day 27. VAS indicates visual analog scale.

A significant main treatment effect of VR was seen on the daily worst experienced pain score (*F* (1, 91.425) = 33.3, *P* < 0.001) (Fig. 3). There was no significant main effect of time (*F* (27,465.3) = 0.481, *P* = 0.99) or treatment-time interaction (*F* (27,465.3) = 0.678, *P* = 0.89). A significant main treatment effect was seen on the daily least experienced pain score (*F* (1, 30.069 = 11.5, *P* = 0.002). There was no significant main effect of time (*F* (27,767.5) = 0.662, *P* = 0.91) or treatment-time interaction (*F* (27,767.497) = 1.279, *P* = 0.157). No differences were seen in scores of pain interference with moving in bed, taking a deep breath or coughing, and sleeping. In the VR group, 47% of patients used opioids at least once in week 1 opposed to 28% in week 4. Opioid use in the control group remained at 37% (Table 3). No change was seen in the use of paracetamol or NSAIDs. The VR group showed a slight but significant improvement on the NEADL subdomain leisure (median VR: 1.50 and control: 0.0), *U* = 51.0, *z* = −3.54, *P* < 0.001, and nonsignificant improvement on the NEADL subdomain domestic (median: 0.50 and 0.0), *U* = 100.5, *z* = −1.88, *P* = 0.06 and HADS depression subscale (median: −1.0 and −1.0), *U* = 125.5, *z* = −0.66, *P* = 0.51. No between-group differences were seen at 4 weeks and 4 months for the pain catastrophizing scale, pain coping and cognition list, brief pain inventory, Oswestry Low Back Pain Disability Index, NEADL subdomains mobility and kitchen, Positive Health Questionnaire, and HADS anxiety subscale (Appendix B, Supplemental Digital Content 2, http://links.lww.com/CJP/A952). Age was not correlated to delta scores for the SF12 physical subscale (*R* = −0.096, *P* = 0.71) and mental subscale (*R* = 0.377, *P* = 0.14).

**TABLE 3:** Number (%) of Participants Reported Have Used Analgesics at Least Once in Week 1 and in Week 4

	Intervention		Control	
	Week 1 (n = 19)	Week 4 (n = 18)	Week 4 (n = 16)	Week 1 (n = 16)
Paracetamol	12 (63)	11 (61)	11 (69)	11 (69)
NSAID	6 (32)	7 (39)	9 (56)	9 (56)
Opioids	9 (47)	5 (28)	7 (37)	7 (37)

NSAID indicates nonsteroidal anti-inflammatory drug.

### Feasibility

Three out of 20 (15%) patients in the VR group reported mild and temporary symptoms of dizziness. No patients discontinued the intervention because of adverse events. During the weekly telephone calls, 1 patient reported not being motivated to adhere to the study protocol, 3 patients reported to have missed 2 out of 7 sessions per week, and 4 patients reported to have missed 1 session per week.

## DISCUSSION

### Main Findings

We did not find an effect of 4 weeks of VR behavioral therapy-based pain management and education intervention on the physical and mental function of patients with nonspecific CLBP referred to a pain clinic. VR seemed to positively affect daily worst and least experienced pain. Opioid use in the VR group was halved. VR was associated with good treatment adherence and minimal and mild side effects.

The limited benefit of VR may be explained by the application itself, patient expectations, a mismatch between the design and evaluation of the VR application, and the execution of the study during the coronavirus disease 2019 (COVID-19) pandemic. This was the first properly designed comparative pilot study wherein this VR application was evaluated for an effect on patient-reported health-related quality of life, mental and physical function, and pain and disability outcomes. Despite a rigorous design together with stakeholders, the application might not yet fully match the needs of patients with CLBP who failed initial physical and pharmacological therapy. The outcomes in the VR group may have been affected by the patient’s expectations for benefit of the VR treatment considering the follow-up treatment they were waitlisted for. Patients with CLBP with a high expectation of improvement are 2.4 times more likely to report improvement in functional limitations, pain intensity, and work participation.^37^ Investigating expectations might have helped explain the different effects of the VR intervention on function and daily pain compared with control. Moreover, it could have revealed a possible mismatch between “patient-considered” compared with “provider-intended” domains of treatment effects. We recently reported in a scoping review that design and evaluation of the same digital technology are often misaligned regarding objectives and user groups, resulting in a limited understanding of the potential of the digital technology and thereby hindering successful implementation.^38^ One could argue that by design the main effect domain of the VR application is the patient’s understanding and “mastering” the pain, which is different from the chosen primary and secondary outcome domains.

Patients in our study had on average 4 to 5 years CLBP with pain scores more than 4 at inclusion, and 40% used opioids. A longer duration of low back pain has been associated with a less favorable prognosis in a meta-analysis with an average baseline pain duration varying from 6 to 12 weeks in the trials.^39^ Thus, possible improvements in our study population might have been smaller than anticipated.

We enrolled patients during the COVID-19 pandemic. It is unknown how the COVID pandemic has impacted the results of this study. Both increased and decreased pain, anxiety, and depression have been described in chronic pain patients.^40^,^41^ Some patients in the current study reported less pain due to decreased social pressure and an altered perspective on health and wellbeing.

An interesting observation in this study is the reduction in opioid use. This reduction was by patient choice and has not been shown in previous studies with behavioral therapy-based VR for CLBP. Confirmation of this observation in larger well-powered studies could have large implications for clinical practice, reducing the need for opioids significantly and thus reducing opioid dependence, including its complications, and health care costs.

Garcia et al^21^ performed a similarly designed randomized controlled study in patients with CLBP, however, with pain reduction as the primary outcome parameter, a larger sample size, and 8 weeks of self-administered treatment. Their VR application reduced pain intensity scores with significant treatment-time interaction and improved physical function and sleep. However, no reduction in opioid use was observed, and similar to our study no change in pain coping and pain catastrophizing. Follow-up data in our series showed no durable effect of the VR intervention on pain. The effects are difficult to interpret in our study due to additional pain interventions during follow-up in both groups. Garcia et al^42^ found a durable treatment effect on pain, however, did not report pain interventions in the follow-up period. There are 2 important methodological differences between Garcia’s study and ours. First, Garcia and colleagues included a convenience sample of participants through chronic pain organizations and social media, whereas we screened all patients referred to a pain clinic. This may have resulted in less bias regarding incentives and motivation to participate in our study. Second, they used an active control group with sham VR, which does not reflect standard care as was administered in our control group. We recently demonstrated in a systematic review of VR use for procedural, acute, and chronic pain, a smaller size effect when the VR intervention was compared with sham VR as opposed to no intervention.^20^

Comparing our study findings with other VR studies in chronic pain has limited value due to other than CLBP patients included, for example, with complex regional pain syndrome,^43^ cancer pain,^44^ fibromyalgia,^45^ and chronic neck pain.^46^ Also, these studies used a VR pain distraction application. We argue whether distraction is the appropriate VR mechanism, by which chronic pain is treated and propose that patient education and pain control by other mechanisms such as presence, embodiment, and interaction may have a more durable effect.^47^

### Strengths and Limitations

This study has several strengths and limitations. This practice-based study was conducted in accordance with the IMMPACT guidelines, which are strongly recommended for chronic pain research.^26^ The successful study conducted during the COVID pandemic, with social distance measures and limited resources, underlines the potential of a safe self-administered home-based VR program for patients with CLBP. Another strength is the intervention design, which is a co-creation with patients, psychologists, educationalists, and software developers. This study might have benefitted from a larger sample size for sufficient power to answer the study question on the effectiveness of this novel VR application. The intensity and duration of the VR intervention might have been inappropriate to establish a significant treatment effect. We advised a minimum of 10 minutes and a maximum of 30 minutes in 1 session per day according to previous publications on VR in general.^17^,^19^ The duration of 4 weeks was according to the expected waiting time for a follow-up visit at the pain clinic. Most patients reported adherence to this advice during the telephone interview, while also reporting that the VR application became monotonous over time.

When compared with standard CBT, the total amount of VR treatment of <5 hours in 4 weeks is rather low. Behavioral therapy is given for a minimum of 6 weeks for 6 to 10 hours in total and is proven to be more effective on pain intensity when used even longer.^48^ Furthermore, studies evaluating a total of <6 hours of CBT have shown no significant decrease in pain intensity.^48^ This intervention was introduced as a self-administered VR program without further guidance on how to apply learned principles in daily life.

It is known from a network meta-analysis of psychotherapy studies that a waiting list setting can induce a nocebo effect with larger effect sizes of treatment compared with no treatment groups outside this setting.^49^ It was hypothesized that patients on the waiting list may unintentionally keep up with disease symptoms to receive the anticipated treatment. Similarly, this may have negatively affected outcomes in our study, particularly in the VR group.

### Clinical Implications and Recommendations

A CBT-based VR intervention might offer a valuable contribution to the multidisciplinary biopsychosocial treatment of CLBP and possibly other chronic pain conditions. This study illustrates that VR pain treatment can be brought close to the patient’s personal environment allowing them to access the treatment at a time and place of their choice. This VR intervention might serve as a tool to narrow the “know-do” gap and help therapists and physicians to apply the psychosocial components of CLBP treatment. VR has the potential to enhance treatment effects through emotional engagement, sense of presence, and personal experiences and might, therefore, have added value over other home-based behavioral therapies.^50^ However, more research and development are needed to investigate how this VR application can be optimally applied as an effective treatment for chronic pain. Specifically for the treatment of CLBP, we believe that the VR application should be integrated into a multidisciplinary treatment with the support of psychologists or trained physiotherapists and take into account the patient’s expectations. Integration in a telehealth solution or combined with physical visits to the pain clinic would help patients to translate learnings to daily life and may increase the effectiveness of VR.^12^,^24^ A recent study of patients rehabilitating from post–COVID-19 conditions showed the feasibility of a multimodal interactive high-intensity physical exercising VR program, supported by community-based physiotherapists.^51^

We hypothesize that the extension of the session and total duration of the intervention while avoiding repetitiveness, could contribute to the success of this VR application. Further intensifying VR use seems possible considering the low rate of minor adverse effects in the current study. Based on the results of this study, no claims can be made about the cost-effectiveness of this VR intervention. Establishing the cost-effectiveness of treatment for chronic pain proves very complex due to the multiple domains of life that chronic pain affects.^52^ However, the VR intervention in this study can be expected to affect a number of factors influencing cost-effectiveness. By bringing therapy to a patient's home, VR might reduce the number of physical visits for a patient while potentially increasing the effectiveness of the multidisciplinary treatment. Also, a VR system is a one-time purchase and can be reused for several patients. VR technology is developing rapidly and VR systems are becoming cheaper and more common, and with that increasingly easier accessible with time. By reducing the pain intensity, it has the potential to aid patients in their activities of daily living and reduce costly disabilities. After this first explorative study, we aim to conduct a large randomized controlled trial investigating (cost) effectiveness in a primary care setting with a multimodal (physical, mental, cognitive-behavioral, and education) VR program for complex CLBP.

## CONCLUSION

We conclude that 4 weeks of a novel self-administered behavioral therapy-based VR program for CLBP does not seem to improve quality of life, but is well tolerated and may positively affect daily pain experience.

## Supplementary Material
