# Supplementary material for: Effect of a Behavioral Therapy-Based Virtual Reality Application on Quality of Life in Chronic Low Back Pain
Source: Clin J Pain. 2023 Apr 1;39(6):278–85. doi: 10.1097/AJP.0000000000001110 (PMC10205123; doi:10.1097/AJP.0000000000001110)
Supplement: Supplementary file 1 [file ajp-39-278-s001.pdf]

Appendix A. Illustrations and descriptions of the modules in the psychological VR application.*

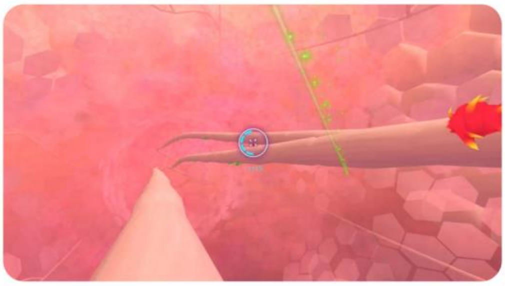

Nerves: Hypnotherapy-based, interactive game in which players have to shoot the threatening stimuli visualised as red monsters racing over nerves.

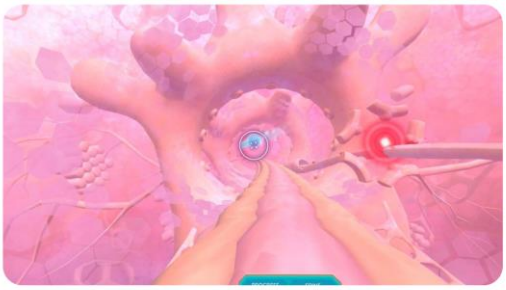

Spinal cord: Mindfulness-based, interactive game in which players have to collect positive neurotransmitters and focus on their respiration rate to turn portals from red into green.

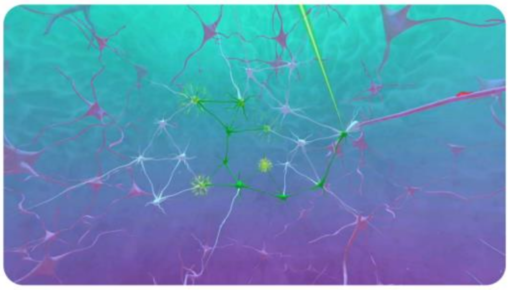

Brain: Interactive game based on ACT and EMDR principles. Players have to switch attention between the pain and the game, helping them to decrease the experienced pain.

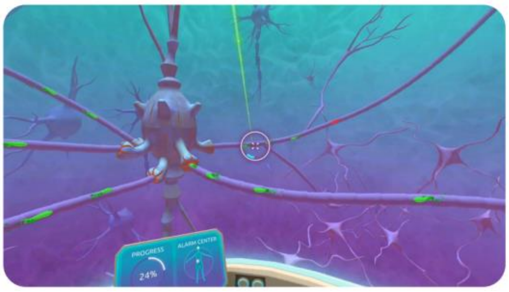

Alarm centre: Interactive game in which patients have to shoot red threatening stimuli into green positive stimuli in order to open their control centre.

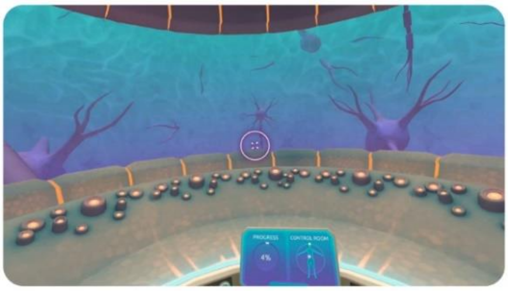

Control room: passive CBT- and mindfulness based game in which patients focus on recognizing and restructuring negative thoughts and connecting with their bodies.

* Reducept Game - Play-through Demonstration - YouTube
