# Supplementary material for: Effect of a Behavioral Therapy-Based Virtual Reality Application on Quality of Life in Chronic Low Back Pain
Source: Clin J Pain. 2023 Apr 1;39(6):278–85. doi: 10.1097/AJP.0000000000001110 (PMC10205123; doi:10.1097/AJP.0000000000001110)
Supplement: Supplementary file 2 [file ajp-39-278-s002.pdf]

Appendix A. Table observed means (SD) for each group for all effectiveness outcome measures at baseline, 4 weeks and 4 months.

	Baseline		4 weeks		4 months	
	VR (n=20)	Control (n=20)	VR (n=19)	Control (n=18)	VR (n=18)	Control (n=16)
SF-12 (0-100)						
- Physical	34.9 (7.5)	32.9 (7.7)	39.1 (6.3)	34.8 (7.1)	38.5 (8.7)	36.8 (7.5)
- Mental	45.6 (7.2)	43.0 (8.9)	48.9 (7.5)	46.0 (10.9)	46.4 (10.1)	46.2 (11.8)
PCS						
- Total (0-52)	21.7 (12.2)	24.7 (7.8)	23.4 (13.8)	25.7 (9.5)	23.9 (12.5)	27.1 (9.7)
- Rumination (0-16)	7.3 (4.5)	9.6 (2.9)	8.2 (4.7)	10.2 (4.1)	9.1 (4.5)	10.2 (3.8)
- Magnification (0-12)	2.6 (2.3)	2.7 (2.4)	4.1 (3.6)	3.2 (2.4)	3.7 (3.0)	3.8 (2.4)
- Helplessness (0-24)	11.8 (6.3)	12.4 (3.5)	11.1 (6.2)	12.9 (4.8)	11.1 (6.5)	13.2 (5.0)
PCCL (0-6)						
- Catastrophizing	3,3 (1,0)	3,7 (0,8)	2,9 (0,9)	3,6 (0,9)	3,3 (0,6)	3,3 (0,9)
- Pain coping	3,5 (0,5)	3,4 (0,9)	3,4 (0,8)	3,1 (0,9)	3,4 (1,0)	3,4 (1,0)
- Internal pain management	3,6 (0,7)	3,3 (1,0)	3,5 (0,9)	3,2 (0,7)	3,6 (0,9)	3,6 (0,7)
- External pain management	3,0 (0,9)	3,1 (0,9)	2,8 (1,0)	2,9 (0,9)	3,1 (0,9)	3,2 (0,9)
BPI, PIS (0-10)	5.9 (1.7)	6.3 (2.0)	4.1 (2.4)	4.8 (2.5)	4.6 (2.1)	5.2 (2.0)
ODI (0-100)	40.1 (19.1)	42.8 (18.8)	32.4 (15.1)	38.8 (17.7)	37.3 (14.5)	40.9 (17.6)
NEADL						
- Mobility (0-18)	13.9 (2.9)	12.4 (4.4)	14.9 (2.9)	12.9 (3.9)	14.1 (4.1)	12.6 (3.5)
- Kitchen (0-15)	13.7 (1.8)	13.8 (1.8)	14.7 (0.8)	13.7 (1.7)	14.0 (2.3)	13.9 (1.6)
- Domestic (0-15)	12.1 (2.9)	12.3 (2.8)	13.4 (2.6)	11.7 (3.0)	11.8 (3.5)	11.5 (2.9)
- Leisure (0-18)	11.9 (3.7)	11.5 (3.7)	14.8 (3.5)	11.4 (3.5)	14.2 (3.8)	11.9 (3.0)
Positive Health						
- Total (0-420)	285.2 (42.4)	265.9 (49.5)	289.1 (67.4)	268.5 (58.1)	194.2 (52.6)	265.6 (65.7)
- Bodily functions (0-70)	33.5 (10.8)	26.6 (13.0)	37.9 (14.1)	29.3 (12.3)	34.5 (15.4)	30.3 (13.3)
- Mental wellbeing (0-70)	51.5 (5.8)	47.3 (10.7)	50.3 (11.0)	46.6 (12.0)	50.9 (9.5)	47.3 (12.2)
- Meaningfullness (0-70)	47.8 (10.2)	41.8 (12.5)	48.5 (10.7)	40.2 (14.7)	50.7 (11.9)	42.1 (12.6)
- Quality of life (0-70)	49.1 (10.2)	46.1 (11.7)	49.0 (12.9)	45.9 (12.2)	50.8 (10.8)	46.4 (12.5)
- Participation (0-70)	53.9 (8.8)	54.4 (8.6)	53.1 (12.4)	53.2 (7.9)	56.0 (6.4)	52.3 (12.8)
- Daily functioning (0-70)	49.5 (8.4)	49.8 (8.6)	50.3 (12.9)	50.5 (9.0)	51.3 (7.9)	47.3 (11.0)
HADS						
- anxiety (0-21)	6.4 (2.8)	9.0 (4.4)	6.6 (4.5)	8.0 (4.1)	6.6 (4.3)	7.4 (3.3)
- depression (0-21)	5.7 (3.5)	8.3 (3.9)	4.7 (3.9)	6.8 (4.4)	6.1 (4.2)	6.0 (4.0)

SF-12: Short Form-12; PCS: Pain Catastrophizing Score; PCCL: Pain Coping and Cognition List; BPI PIS: Brief Pain Inventory, Pain Interference Score; ODI: Owestry Low Back Pain Disability Index; NEADL: Nottingham Extended Activities of Daily Living; HADS: Hospital Anxiety and Depression Scale.
